# Proteomic and Molecular Assessment of the Common Saudi Variant in *ACADVL* Gene Through Mesenchymal Stem Cells

**DOI:** 10.3389/fcell.2019.00365

**Published:** 2020-01-10

**Authors:** Ahmad Alfares, Majid Alfadhel, Ahmed Mujamammi, Batoul Alotaibi, Sarah Albahkali, Mohammed Al Balwi, Hicham Benabdelkamel, Afshan Masood, Rizwan Ali, Amani Almuaysib, Saeed Al Mahri, Sameer Mohammad, Ibrahim O. Alanazi, Assim Alfadda, Saleh AlGhamdi, Bahauddeen M. Alrfaei

**Affiliations:** ^1^Department of Pediatrics, College of Medicine, Qassim University, Al-Qassim, Saudi Arabia; ^2^Department of Pathology and Laboratory Medicine, King Abdulaziz Medical City, MNGHA, Riyadh, Saudi Arabia; ^3^Division of Genetics, Department of Pediatrics, King Abdullah Specialized Children Hospital, King Abdulaziz Medical City, MNGHA, Riyadh, Saudi Arabia; ^4^Medical Genomics Research Department, King Abdullah International Medical Research Center, MNGHA, Riyadh, Saudi Arabia; ^5^College of Medicine, King Saud Bin Abdulaziz University for Health Sciences, MNGHA, Riyadh, Saudi Arabia; ^6^Unit of Clinical Biochemistry/Medical Biochemistry, Department of Pathology, College of Medicine, King Saud University, Riyadh, Saudi Arabia; ^7^Stem Cells Unit, Department of Cellular Therapy, King Abdullah International Medical Research Center, MNGHA, Riyadh, Saudi Arabia; ^8^Proteomics Resource Unit, Obesity Research Center, College of Medicine, King Saud University, Riyadh, Saudi Arabia; ^9^Medical Core Facility and Platforms Department, King Abdullah International Medical Research Center, MNGHA, Riyadh, Saudi Arabia; ^10^Department of Experimental Medicine, King Abdullah International Medical Research Center, MNGHA, Riyadh, Saudi Arabia; ^11^National Centre for Biotechnology, Life Science and Environment Research Institute, King Abdulaziz City for Science and Technology, Riyadh, Saudi Arabia; ^12^Clinical Research Department, Research Center, King Fahad Medical City, Riyadh, Saudi Arabia

**Keywords:** VLCAD, long-chain fatty acid, mitochondria, mesenchymal stem cells, proteomic, metabolic disorder

## Abstract

Very-long-chain acyl-coenzyme A dehydrogenase (VLCAD) is a coenzyme encoded by *ACADVL* that converts very-long-chain fatty acids into energy. This process is disrupted by c.65C > A; p.Ser22^∗^ mutation. To clarify mechanisms by which this mutation leads to VLCAD deficiency, we evaluated differences in molecular and cellular functions between mesenchymal stem cells with normal and mutant VLCAD. Saudi Arabia have a high incidence of this form of mutation. Stem cells with mutant VLCAD were isolated from skin of two patients. Metabolic activity and proliferation were evaluated. The Same evaluation was repeated on normal stem cells introduced with same mutation by CRISPR. Mitochondrial depiction was done by electron microscope and proteomic analysis was done on patients’ cells. Metabolic activity and proliferation were significantly lower in patients’ cells. Introducing the same mutation into normal stem cells resulted in the same defects. We detected mitochondrial abnormalities by electron microscopy in addition to poor wound healing and migration processes in mutant cells. Furthermore, in a proteomic analysis, we identified several upregulated or downregulated proteins related to hypoglycemia, liver disorder, and cardiac and muscle involvement. We concluded experimental assays of mutant *ACADVL* (c.65C > A; p.Ser22^∗^) contribute to severe neonatal disorders with hypoglycemia, liver disorder, and cardiac and muscle involvement.

## Introduction

Fatty acid oxidation disorders are a collection of recessive disorders triggered by an insufficiency in enzymes involved in β-oxidation or the movement of long-chain fatty acids into the mitochondria. Body energy is produced by mitochondria by a complex mechanism involving the fatty acid beta oxidation pathway, which delivers an important supply of energy; long-chain fatty acids require carnitine transport ability to enter the mitochondria in order to produce ketone bodies, which provide a form of energy for the brain, heart, muscle, kidney, and other tissues (Sun and Merritt, 2015; Leslie et al., 2018).

*ACADVL* encodes very-long-chain acyl-CoA dehydrogenase (VLCAD) and mutations in this gene can result in VLCAD deficiency (OMIM #201475). Null alleles are associated with a severe early onset phenotype, whereas missense or in-frame deletion alleles are often, but not always associated with a milder, late-onset form of VLCAD deficiency (Miller et al., 2015). *ACADVL* interacts with esters of long-chain and very-long-chain fatty acids (McAndrew et al., 2008). Cardiolipin binding is regulated by reversible lysine acylation; this mechanism is predicted to apply to other metabolic proteins that localize to the inner mitochondrial membrane (Zhang et al., 2015) and could explain hypertrophic cardiomyopathy in mice (Chen et al., 2016). However, information about the effect of VLCAD deficiency is either lacking (e.g., in stem cells, lung cells, and neurons) or incomplete (e.g., in myocytes and liver cells) (Aoyama et al., 1995). In mice with VLCAD deficiency, there is little to no protein hyperacetylation in the liver, suggesting that VLCAD is necessary for protein acetylation in the species (Pougovkina et al., 2014).

Symptomatic and asymptomatic neonates are identified through newborn screening (NBS) using dried blood spots for a comprehensive acylcarnitine analysis by tandem mass spectrometry (McHugh et al., 2011). Diagnosis depends on an analyses of the plasma acylcarnitine profile and urine organic acids, followed by genetic or enzymatic measurements for confirmation (Hale et al., 1985; Spiekerkoetter et al., 2009; Wilcken, 2010; Bouvier et al., 2016). The prevalence of this disorder in Saudi Arabia is not known; however, published data from an institutional NBS program have shown that VLCAD is one of the most commonly identified disorders, with an incidence of 1:37,000 individuals at the Ministry of National Guard Health Affairs (Alfadhel et al., 2016). One founder loss-of-function variant, c.65C > A (p.Ser22^∗^), in *ACADVL* accounts for around 80% of all identified variants associated with a VLCAD deficiency in the Saudi population (Alfadhel et al., 2016). In worth nothing that VLCAD deficiency was found in multiple countries such as China, Japan, Vietnam, and India (Shibata et al., 2018). The nonsense variant c.65C > A (p.Ser22^∗^) in *ACADVL* is predicted to cause a loss of function of the protein by creating a premature stop codon. Currently, there are no treatments for VLCAD. Triheptanoin does not prevent the progression of cardiac dysfunction in VLCAD-deficient mice (Tucci et al., 2017). Management is based on the signs and symptoms present in each patient.

In this study, we characterized molecular and proteomic differences between *ACADVL*-mutated human mesenchymal stem cell and the normal mesenchymal stem cell line, Hs27, used as a control. We compared these results to those obtained using patient primary fibroblasts. The goal of this study was to improve our understanding of the commonly identified c.65C > A; p.(Ser22^∗^) *ACADVL* variant in the Saudi population.

## Results

### Skin Stem Cells From VLCAD-Deficient Patients Have Reduced Metabolic Activity

**FIGURE 1:**
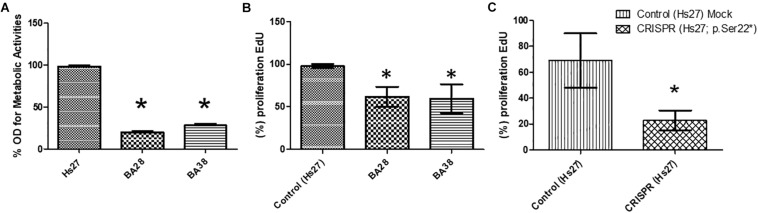
Defective proliferation of VLCAD-deficient cells. **(A)** Metabolic activity (MTT) was lower in patient cells than in control cells (Hs27). **(B)** Based on an EdU assay, proliferation was lower in patient cells than in control cells. **(C)** In normal cells (Hs27), the *ACADVL* mutation was introduced using CRISPR technology. Mutant cells exhibited reduced proliferation in reference to mock controls. Asterisk (^∗^) represents significance at *P* < 0.05.

Skin stem cells from patients (BA-28 and BA-38) were used to measure metabolic activity and viability by MTT assays. Patient-derived cells showed significantly lower metabolic activity than that of H27 cells, which are normal mesenchymal stem cells. Both samples BA-28 and BA-38 had 65–85% less metabolic activity than that of Hs27 cells (Figure 1A; *P* < 0.05). MTT is an indicator of proliferation and expansion; cell proliferation was evaluated more directly using the EdU proliferation assay. Patient samples showed significantly lower rates of proliferation than those of control cells (Figure 1B; *P* < 0.05).

To study the direct impact of the *ACADVL* mutation (c.65C > A; p.(Ser22^∗^), we used CRISPR gene editing technology to induce the specific mutation in Hs27 cells. Interestingly, mutant Hs27 cells had a significantly reduced proliferation rate (by 25–50%) compared to that of normal Hs27 cells (Figure 1C; *P* < 0.05).

### Decreased Wound Healing Ability in Patient Cells

**FIGURE 2:**
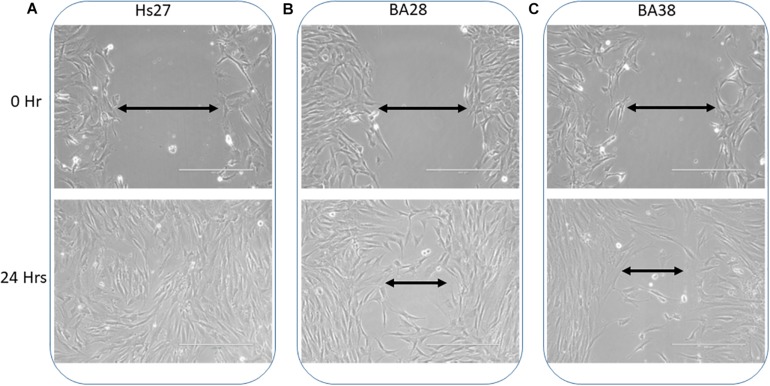
Reduced wound healing ability in ACADVL-mutated cells. **(A)** Normal cells (Hs27) showed rapid wound healing within 24 h. **(B)** Patient (BA28) cells exhibited less competent wound healing ability than that of normal cells within 24 h. **(C)** Patient (BA38) cells show reduced wound healing ability compared to normal cells after 24 h.

Monolayers of patient skin mesenchymal stem cells exhibited a decreased wound healing ability compared to that of normal cells within 24 h (Figures 2A–C; *P* < 0.05). Normal cells had a 40% greater ability to heal and close wounds (Figure 2A). Cells of both patients, BA-28 and BA-38, had lower wound healing ability than that of normal cells (Figures 2B,C).

### Glucose Uptake Defect in Patient Cells

**FIGURE 3:**
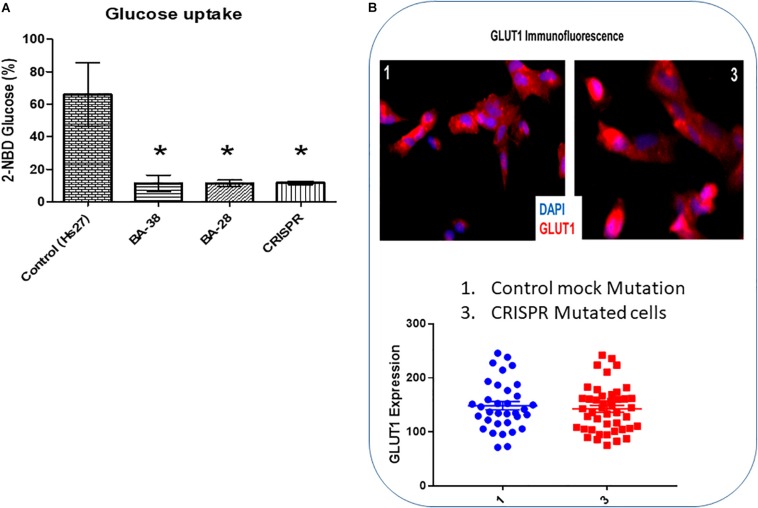
Glucose uptake is inhibited in cells with mutant *ACADVL*. **(A)** Evaluation of glucose uptake shows severe inhibition (80–90%) in patient cells compared to normal cells. In addition, introducing the *ACADVL* mutation into Hs27 cells resulted in the same level of glucose uptake inhibition. **(B)** Evaluation of GLUT1 activity; both CRISPR-mutated cells and control cells had the same level of GLUT1 activity.

The glucose uptake rate was significantly lower in patient cells (BA-28 and BA-38) (*P* < 0.01) than in normal cells (Hs27) (Figure 3A). Moreover, CRISPR-mutated normal cells (Hs27) with c.65C > A (p.Ser22^∗^) exhibited significantly less glucose uptake than that of mock-mutated cells (Figure 3A; *P* < 0.01). The levels of GLUT1 expression in these cells, as determined by quantitative immunofluorescence, were the same in mock and p.Ser22^∗^-mutated cells (Figure 3B).

### Abnormal Mitochondria in Patient Specimens

**FIGURE 4:**
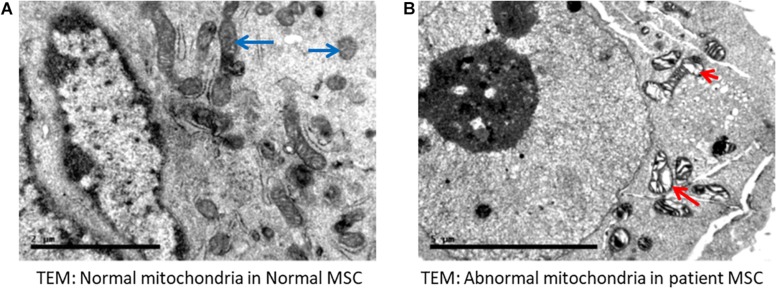
Electron microscopy indicated abnormal mitochondria. **(A)** Normal mesenchymal stem cells (MSC) showing normal mitochondria (long blue arrows). **(B)** Representative cells from a patient with VLCAD deficiency showing abnormal mitochondria with cyst formation (short red arrow). Long arrow shows abnormal mitochondrial morphology, in addition to fewer and disarrayed cristae. Long red arrow indicates an area of abnormal electron density with ruptured membranes or large vacuoles.

High magnification screening of mitochondrial structures showed serious defects in patient samples compared to normal samples. Cells isolated from both patients had mitochondrial cysts detected at 40,000× magnification (Figure 4B). Additionally, an abnormal mitochondrial morphology with a low electron density was detected in patient cells. Moreover, electron micrography of patient cells showed mitochondrial membrane ruptures or large vacuoles (Figure 4B).

### Identification of Differentially Abundant Proteins

**FIGURE 5:**
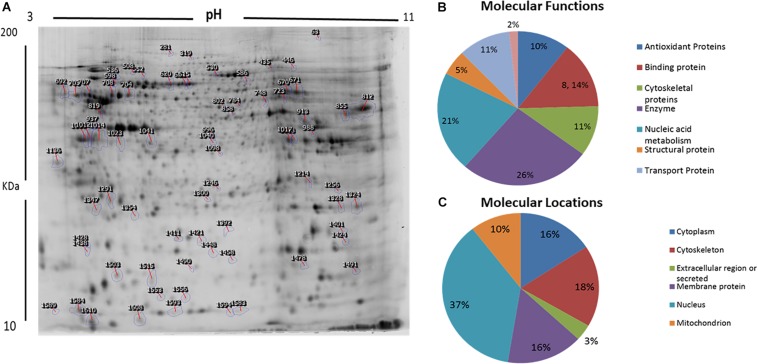
Representative image of protein spots from VLCAD deficiency samples and a comparative analysis. **(A)** Numbered spots indicate those that were differentially expressed (over 1.5-fold change, *P* < 0.05) and were successfully identified by MALDI-TOF/TOF. **(B)** Comparative analysis (%) of proteins categorized into groups according to their function. **(C)** Comparative analysis (%) of identified proteins categorized into groups according to their cellular location.

We used 2D-difference gel electrophoresis (2D-DIGE) with technical replicates to obtain reproducible spot patterns for all samples from patient cells and the control line, Hs27. Approximately 1100 spots were mapped to the gels (Supplementary Figures S1A–C). Proteins that were differentially expressed between the control and patient cells were visualized in gels (yellow spots in Supplementary Figure S1D). We show a representative 2D-DIGE gel from the set of pairwise comparisons between groups in Figure 5. Spots with differences in intensity between groups were detected (Figure 5A). The spot patterns were reproducible across six gels and therefore alignment and further analysis was possible. A total of 72 protein spots showing significant increases or decreases in expression between the mutated and control cells were detected. In some cases, variants of the same protein were found at several locations on the gel. We found that 57 of the 72 spots were unique protein sequences by MALDI-TOF mass spectrometry and matched to entries in the SWISS-PROT database by Mascot with high confidence. Of these 57 spots, 37 spots were upregulated and 20 were downregulated in patient cells when compared with control cells. The 57 protein spots identified as differentially abundant are summarized in Figure 5, Table 1, and Supplementary Table S1.

**TABLE 1:** Proteomic analysis represent a list of significant proteins differentially expressed proteins between patient’s samples and controls.

**Accession no.**	**Protein name**	**Fold change**	***P* value**	**VLCAD/Control ratio**
P04406	Glyceraldehyde-3-phosphate dehydrogenase	2.1	0.00001	Up
Q6XPS3	Phosphotidylinositol 3,4,5-triphosphate 3-phaosphate	1.6	0.004	Up
P22392	Nucleoside diphosphate kinase B	2.2	0.005	Up
P14618	Pyruvate kinase PKM	1.8	0.02	Up
P63241	Eukaryotic translation initiation factor 5A-1	1.8	0.008	Up
P38646	Stress-70 protein, mitochondrial	2.3	0.008	Down
Q99733	Nucleosome assembly protein 1-like 4	1.9	0.04	Dawn
P63241	Eukaryotic translation initiation factor 5A-1	1.5	0.002	Up
Q9BY41	Histone deacetylase 8	1.7	0.01	Up
P436S6	26S proteasome regulatory subunit6B	2.6	0.01	Down
Q9P1Z9	Coiled-coil domain-containing protein 180	1.8	0.01	Down
PS2907	F-actin-capping protein subunit alpha-1	1.9	0.05	Up
P11142	Heat shock cognate 71 kDa protein	1.7	0.04	Up
P43686	25S proteasome regulatory subunit 6B	2.6	0.01	Down
P68104	Elongation factor 1 alpha 1	1.54	0.03	Down
Q07021	Complement component 1 Q subcomponent-binding protein, mitochondrial	2.2	0.04	Down
Q06S30	Peroxiredoxin-1	1.7	0.04	Down
P07910	Heterogeneous nuclear nbonucleoproteins C1/C2	1.7	0.03	Up
P12270	NucleoproteinTPR	1.5	0.01	Up
P07437	Tubulin beta chain	1.9	0.015	Up
P07237	Protein disulfide-isomerase	1.7	0.08	Up
P62805	Histone H4	2.5	0.08	Up
PO8238	Heat shock protein	1.6	0.08	Up
PD4406	Glyceraldehyde-3-phosphate dehydrogenase	1.8	0.02	Up
P0674S	Nucleophosmin	1.5	0.02	Up
P63261	Actin, cytoplasmic 2	1.5	0.02	Up
Q14204	Cytoplasmic dynein 1 heavy chain 1	1.7	0.02	Up
P10809	60 kDa heat shock protein, mitochondrial	1.9	0.021	Up
P48047	ATP synthase subunit O, mitochondrial	1.5	0.028	Down
P10809	60 kDa heat shock protein, mitochondrial	1.6	0.024	Up
Q9NZJ6	Ubiquinone biosynthesis Omethyltransferase, mitochondrial	1.5	0.03	Up
P68104	Elongation factor 1-alpha 1	2.8	0.03	Down
P11142	Heat shock cognate 71 kDa protein	1.8	0.05	Up
P13639	Elongation factor 2	1.8	0.03	Down
P25705	ATP synthase subunit alpha, mitochondrial	2.5	0.03	Down
P63261	Actin, cytoplasmic 2	1.8	0.03	Up
P0674S	Nucleophosmin	2.2	0.04	Up
P53396	ATP-citrate synthase	1.7	0.04	Down
Q9UI30	Multifunctional methyltransferase subunit TRM112-like protein	1.6	0.03	Up
P07741	Adenine phosphoribosyl transferase	1.8	0.02	Up
Q96KK5	Histone H2Atype 1-H	1.8	0.02	Up
P13639	Elongation factor 2	1.5	0.03	Down
QSWVF1	Protein 0SCP1	1.7	0.05	Up
P14618	Pyruvate kinase PKM	1.7	0.037	Up
P12883	Myosin-7	2.2	0.037	Up
P08670	Vimentin	1.9	0.04	Down
Q13347	Eukaryotic translation initiation factor 3 subunit 1	1.7	0.04	Up
P54709	Sodium/potassium transporting ATPase subunit beta-3	2.3	0.04	Down
Q9Y230	RuvB-like 2	2.7	0.05	Down
P0867D	Vimentin	1.7	0.05	Down
P06576	ATP synthase subunit beta, mitochondrial	1.62	0.05	Down
P35908	Keratin, type II cytoskeletal 2 epidermal	1.8	0.03	Up
Q8J015	60S ribosomal protein LI3a	2.7	0.03	Up
Q14697	Neutral alpha-glucosidase AB	1.9	0.03	Down
P00441	Superoxide dismutase [Cu–Zn]	1.8	0.04	Up
P12883	Myosin-7	1.8	0.04	Up

### Classification of Key Proteins Based on Function

GO and the UNIPROT system were used to identify overrepresented molecular functions and cellular components assigned to the differentially expressed proteins. The main functional categories identified in this analysis were enzyme, nucleic acid metabolism, binding proteins, and cytoskeletal proteins (Figure 5B). The locations of these proteins were also obtained (Figure 5C).

### PCA

**FIGURE 6:**
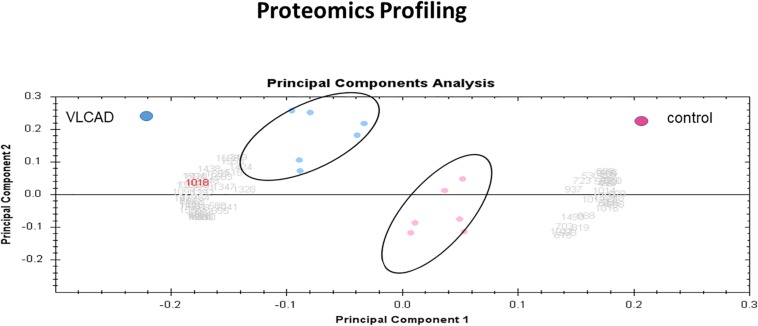
PCA plot of the two first principal components (VLCAD and control). The two components combined explained 82% of the overall variation. Colored dots and numbers are representative of gels and spots, respectively.

Multivariate analyses of protein abundance data were performed using Progenesis SameSpots. The gel images were grouped such that technical replicates (three samples with mutant VLCAD and three control samples) formed the two groups. The data were filtered so that only 57 spot features exhibiting statistically significant (ANOVA, *P* < 0.05) changes in abundance, present on all gel images, and identified by MS were considered. In these analyses, the two groups formed distinct clusters based on the presence of disease (Figure 6). The hierarchical cluster analysis of spots with increased abundance indicated 37 proteins that were upregulated in patients with VLCAD deficiencies (Supplementary Figure S2).

### Protein–Protein Interaction Networks

**FIGURE 7:**
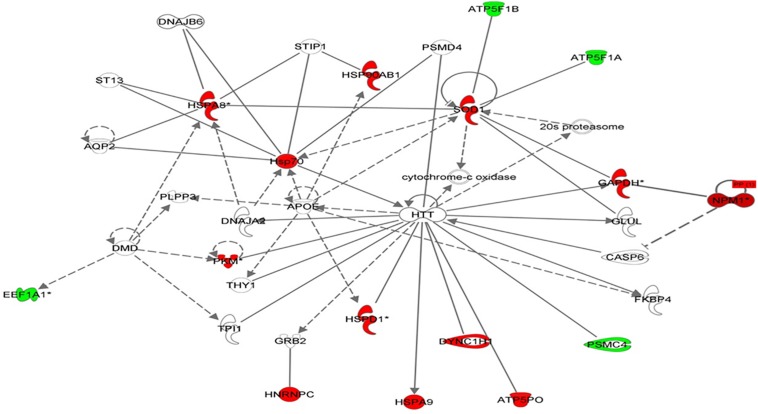
Schematic representation of the most significant IPA networks identified in VLCAD deficient patients. IPA analysis of functional interaction networks of differentially regulated proteins in cells with a VLCAD deficiency compared to control cells. The highest reported score of 30 was related to “post-transition modification, protein folding, neurological disease,” showing HTT and ApoE as central nodes. Green and red correspond to down- and upregulated proteins, respectively. Non-colored nodes indicate potential targets functionally coordinated with the differential proteins. Solid lines indicate direct molecular interactions and dashed lines represent indirect relationships.

Using Ingenuity Pathway Analysis (IPA), interaction networks were generated for the proteins exhibiting differential expression. The highest scoring network incorporated 16 out of the 57 focal molecules (Figure 7).

## Discussion

There are three clinical forms of VLCAD deficiency (OMIM #201475), a severe form with neonatal onset and a high rate of mortality due to cardiomyopathy, an intermediate form with hypoketotic hypoglycemia and childhood onset, and an adult-onset form characterized by skeletal involvement and exercise-induced rhabdomyolysis. In this study, the common form in Saudi Arabia, i.e., the most severe form with neonatal onset, was evaluated by studying the most frequent causal variant (c.65C > A; p.(Ser22^∗^)) and the impact of this variant was linked to mitochondrial metabolism, liver, heart, and muscle function from molecular point of view.

Skin mesenchymal stem cells from two patients (BA28 and BA38) were studied with respect to metabolic activity. Both samples showed reduced metabolic activity compared to that of normal, commercially available mesenchymal stem cells (Hs27) (Figure 1A). Furthermore, significantly lower proliferation was found in patient cells than in Hs27 cells, as determined by EdU assays (Figure 1B). The highly reduced metabolic activity could explain the severe symptoms associated with this variant. These observations were confirmed by introducing the c.65C > A mutation using CRISPR technology to the genomes of Hs27 cells; the mutant cells showed a 30% reduction in proliferation, similar to the reduction observed in patient cells (Figure 1C). This extensive reduction in proliferation could explain the early and severe onset of cardiac and liver diseases in these patients.

Defects in GLUT1, encoded by *SLC2A1*, are linked to several disorders, including Dystonia 9 (MIM 601042), GLUT1 deficiency syndrome 1 (MIM 606777), GLUT1 deficiency syndrome 2 (MIM 2612126), stomatin-deficient cryohydrocytosis with neurologic defects (MIM 608885), Susceptibility to idiopathic generalized epilepsy (MIM 614847), and GLUT1 deficiency syndrome 1 and 2. Patient cells and CRISPR-mutated cells both exhibited 80–90% reductions in glucose uptake ability compared to that of controls. These results suggested that there were systemic alterations in glucose uptake and migration. However, normal GLUT1 expression was found in both mutant and control cells, suggesting that GLUT1 is not related to the glucose uptake deficiency in skin mesenchymal stem cells with mutant VLCAD (Figures 3A,B). GLUT1 expression in other tissues of patients with VLCAD deficiencies, like CSF, might be defective. In addition, evaluating other GLUT proteins (i.e., GLUT2–8) may provide insight into the mechanism underlying the glucose uptake deficiency.

Proper liver development early in life requires normal cell migration ability (Krawczyk, 1971; Sosa-Pineda et al., 2000). Patients with VLCAD deficiencies develop liver disorders in the first 2 years of life (Alfadhel et al., 2016). Wound healing in mutated VLCAD cells was 40% slower compared to that in Hs27 cells (Figures 2A–C). This suggests that a liver abnormality in early development may be related to reduced migration ability.

Electron microscopy of mitochondria in patient cells revealed structural abnormalities with cysts and abnormal appearances, in addition to a low electron density and disarranged cristae in comparison to normal cells, supporting the mitochondrial defect in VLCAD deficiencies (Figures 4A,B).

In a proteomic analysis, we identified 57 differentially expressed proteins between patient samples and controls (Table 1). These proteins were involved in the following functional categories: 10% antioxidant, 8% binding, 11% cytoskeletal, 5% structural, 26% enzymes, 21% nucleic acid metabolism, and 11% transport proteins (Figure 5B). Surprisingly, considering the observed defects in the mitochondria and the classification of VLCAD deficiencies as mitochondrial disorders, most of the affected proteins were located outside mitochondria (37% in the nucleus, compared to only 10% in mitochondria) (Figure 5C). Thirty-seven proteins were upregulated in patients with VLCAD deficiencies. Glyceraldehyde-3-phosphate dehydrogenase (GAPDH, UniProtKB P04406), which was upregulated, is closely linked to ACADVL and is involved in the glycolysis and gluconeogenesis pathways, explaining hypoglycemia in patients. Additionally, we found that histone deacetylase 8 is upregulated in patient-derived mesenchymal stem cells, suggesting the inhibition of protein acetylation. A previous study has linked a VLCAD deficiency to a lack of protein hyperacetylation in mouse livers (Pougovkina et al., 2014). Accordingly, we expected patients with VLCAD deficiencies to exhibit reduced acetylation in the liver. Histone H4 and Histone H2A type 1-H were upregulated in our data; these proteins regulate the structure of chromatin and nucleosomes in humans, respectively. These events suggest that there is competition to maintain homeostasis of these structures.

Cardiomyopathy and sudden death have also been reported in patients with VLCAD deficiencies (Sun and Merritt, 2015). Our proteomic data show that both Elongation Factor 1-alpha 1 and Elongation Factor 2 are downregulated in patient cells. These proteins are downregulated in myocardial infarction and are involved in heat shock responses (Horman et al., 2003; Clyne et al., 2014; Vera et al., 2014). Moreover, heat shock proteins (Stress-70 protein, mitochondrial, Heat shock protein, and 60 kDa heat shock protein, mitochondrial) were upregulated in patient samples. The upregulation of heat shock proteins indicates active stress at the molecular level and this stress is likely widely distributed across internal organs, with the potential to lead to cardiomyopathy and sudden death from cardiac arrythmia.

Rhabdomyolysis is associated with VLCAD disease (Voermans et al., 2006). Normal muscle physiology maintains ion channel (Na^+^, K^+^, and Ca^2+^) homeostasis. Muscle cells contract by actin-myosin cross-linking. These procedures are dependent on adequate energy. Therefore, the disruption of these procedures may damage ion channels, injure myocytes, reduce ATP, and disrupt the intracellular electrolyte concentration balance (Torres et al., 2015). Our proteomics data show that Sodium/potassium-transporting ATPase subunit beta-3 protein is inhibited in VLCAD-mutated cells. This protein is responsible for establishing and maintaining the electrochemical gradients of Na and K ions around the plasma membrane. In addition, ATP synthase subunit beta, mitochondrial protein was downregulated in VLCAD-mutated cells. This protein is involved in the utilization of an electrochemical gradient of protons across the mitochondrial inner membrane. These proteins contribute to rhabdomyolysis.

## Conclusion

We present the first molecular and functional analyses of the founder variant c.65C > A; p.(Ser22^∗^) in *ACADVL* in the Saudi population, which is associated with a severe neonatal onset disorder. It worth noting that both patients (BA-28 and BA-38) have passed away. With a limitation of only two patients we detected lower metabolic activity, proliferation, wound healing, and migration in mutant cells than control cells. Furthermore, in a proteomic analysis, we identified several upregulated or downregulated protein to support suggestion for hypoglycemia, liver disorders, cardiac and muscle involvement. However, the mechanisms by which deacetylation enzymes are upregulated in VLCAD deficiency are unclear. Furthermore, additional studies of glucose uptake and GAPDH pathways may provide important insight into glucose alterations in cells and hypoglycemia in patients.

## Materials and Methods

### Human Subjects

Ethical approval for clinical and laboratory participation was obtained from King Abdullah International Medical Research Center (KAIMRC) IRB in Riyadh, Saudi Arabia under protocol number RC16/129/R. Blood was collected from subjects and their parents in EDTA tubes for DNA extraction and variant confirmation. Skin biopsy was used to isolate and culture mesenchymal stem cells collected from affected individuals.

### Tissue Collection and Mesenchymal Stem Cell Isolation

Two individuals were enrolled in this study (BA28 and BA38). A dermal punch biopsy was collected from each patient and transferred to the research laboratory in medium containing advanced RPMI (12633012; Gibco, Gaithersburg, MD, United States) and 1% antibiotic-antimycotic (15240-062; Gibco) solution. The tissues were left in media for 1–2 h after collection. Skin mesenchymal stem cells were then isolated separately using the explant method, as previously described (Vangipuram et al., 2013). In brief, fat was removed from the dermal biopsies and the tissues were chopped and plated in a 6-well plate (three pieces per well). The cells were left undisturbed for 4–6 days in conditioning medium (growth specific media) composed of advanced RPMI (12633012; Gibco), 5% fetal bovine serum (FBS, 10099141; Gibco), 20 pg of rh-FGF (CC-4065J; Lonza, Basel, Switzerland), 0.1% insulin (51500056; Gibco), and 1% antibiotic–antimycotic solution. The conditioning medium was changed every 3 days until fibroblasts or dermal mesenchymal stem cells appeared. Validation was done to make sure that mesenchymal stem cells were isolated. The isolated dermal mesenchymal stem cells were differentiated into neurons to insure multipotent ability in patient cells and Hs27 cell line, Supplementary Figures S3–S6. Mesenchymal stem cells markers (CD90, CD105, CD144) were validated on patients’ cells, Supplementary Figure S4. Hs27 cell line was negative on those markers even though it was able to differentiate in to neurons. Similar dermal fibroblasts isolated from skin was described previously (Alfadhel et al., 2018). It worth noting that both patients (BA-28 and BA-38) have passed away.

### MTT Metabolic Activity Assay

Mesenchymal stem cells (BA28 and BA38) obtained from the skin of patients and normal, control cells (Hs27; CRL-1634^TM^, ATCC, Manassas, VA, United States) were inoculated at 5000 cells per well in 96-well plates with 10% FBS at 37°C overnight. On the next day, all cells were starved with DMEM (Cat #21885025; Thermo Fisher Scientific, Waltham, MA, United States) containing 1% FBS and incubated at 37°C overnight. All cells were nourished with complete growth specific media on the assay day. The patient cells were then compared with control cells (Hs27) using the MTT Cell Proliferation Assay (Cat #V13154; Thermo Fisher Scientific), following the manufacturer’s protocol. Signals were read using SpectraMax Reader software (SoftMax Pro v.6.2.1).

### BrdU Cell Proliferation Assay (Click-iT EdU)

All cells (BA28, BA38, and Hs27) were inoculated at 2000 cells per well in 96-well plates with DMEM and 10% FBS at 37°C overnight. On the next day, all cells were starved in DMEM containing 1% FBS and incubated at 37°C overnight. All cells were nourished with complete growth specific media on the assay day. Patient and Hs27 cells were compared using the Click-iT EdU Microplate Assay (Catalogue #V13154; Thermo Fisher Scientific), following the manufacturer’s protocol. The signal was read using SpectraMax M5 Reader software (SoftMax Pro v.7.0.2).

### Scratch (Wound Healing) Assay

Monolayers of skin mesenchymal stem cells of patients were grown in advanced RPMI supplemented with 5% FBS. Then, they were seeded into a 6-well tissue culture plate at 250,000 cells/well and incubated at 37°C. After 24 h, cells reached ∼70–80% confluence as a monolayer. Without changing the medium, the monolayer was lightly and deliberately scratched using a sterilized 200-μl pipette tip across the midpoint of the well. The resulting gap distance therefore was equal to the outer diameter of the end of the tip. After making a straight-line scratch in a single direction, the wells were washed two times with the medium to eliminate the non-adherent cells. Then, the medium was replaced. On the next day, the distance of the unclosed gap was measured.

### CRISPR Transfection Assay

A custom CRISPR kit for *ACADVL* c.65C > A p.(Ser22^∗^), CRISPR pCas-Guide-EF1a-GFP, NM_001033859.2 (*ACADVL*): c.65C > A p.(Ser22^∗^), was used. The modified sequence was (ACCGGGCCGGCACTGAACCCCACTCCCCACAGCT[A]GC GGCTCACGGCGCTCCTGGGGCAGCCCCGGCCCG) in pCas-Guide-EF1a-GFP. For transfection, GenMute Reagent (Cat #SL100568, SignaGen, Rockville, MD, United States) was used. Normal cells (200,000 cells) were inoculated in a T25-flask in 10% FBS at 37°C overnight. On the next day, the cells were starved with 1% FBS for 24 h. On day 3, the VLCAD mutation was introduced into normal cells (Hs27) using GenMute Reagent and the CRISPR kit. The GenMute Transfection working solution was prepared by adding the CRISPR construct with the mutation. A second working solution was also prepared using normal DNA as a control. All flasks were incubated for 4 h at 37°C after transfection. Then, media were replaced with fresh Advanced RPMI 1640 conditioned medium, which was also used in the isolation step.

### Glucose Uptake Assay

Patient, CRISPR-mutated (Hs27), and Normal (Hs27) cells were plated separately in a 96-well plate and starved overnight with 1% FBS at 37°C. On the next day, the cells were washed twice with glucose-free DMEM and incubated in the same medium for 2 h. Glucose uptake was measured using the Glucose Uptake analysis Kit (Cayman Chemicals, Ann Arbor, MI, United States) following the manufacturer’s protocol. Briefly, cells were incubated with 100 μg/mL fluorescent 2-*N*-7-Nitrobenz-2-oxa-1, 3-diazol-4-yl-Amino-2-Deoxyglucose (2-NBDG) in glucose-free medium for 1 h. The cells were then washed with assay buffer three times and analyzed immediately. 2-NBDG picked up by the cells was measured using the Tecan Infinite 200 Pro Fluorimeter (Tecan Group Ltd., Männedorf, Switzerland) with fluorescent filters designed to detect fluorescein (excitation/emission = 485/535 nm). The blank control cells were not incubated with 2-NBDG.

### Immunofluorescence Analysis of GLUT1 Expression

A total of 5000 cells grown on cover slips were washed with PBS and fixed with 4% formaldehyde. The cells were incubated with an anti-glut1 antibody (Cat #PA1-46152; Thermo Fisher Scientific) in PBS containing 5% FBS and 0.05% Triton for 30 min at 37°C. The cells were then washed four times with PBS and incubated with fluorescein isothiocyanate (FITC)-conjugated antibodies for 30 min. The cells were then washed four times and images were obtained using the EVOS FL Auto Imaging System (Thermo Fisher Scientific). Images were used to quantify glucose transporter 1 (GLUT1) expression using MetaMorph (Molecular Devices, San Jose, CA, United States).

### Transmission Electron Microscopy

BA33, BA28, and Hs27 cells were propagated, harvested, and pelleted in Eppendorf tubes. Cells were then fixed in 4% glutaraldehyde (Electron Microscopy Sciences [EMS], Hatfield, PA, United States) for 2 h and supplemented with 1% osmium tetroxide (EMS) for 1 h. Then, the samples were dehydrated using 70% ethanol for 10 min, 100% ethanol for 10 min, 100% ethanol for 15 min, and 2 washes in 100% propylene oxide (Merck KGaA, Darmstadt, Germany) for 15 min. Infiltration was performed using a mixture of EMbed 812 One-step Single Mix Formula composed of 20 mL of EMbed 812 (EMS), 16 mL of dodecyl succinic anhydride (DDSA) (EMS), 8 mL of methyl-5-norbornene-2,3-dicarboxylic anhydride (NMA) (EMS), and 0.66–0.88 mL of 2,4,6-tri(dimethylaminomethyl) phenol (DMP-30) (EMS). The cells were then soaked in a 1:1 solution of propylene oxide: embedding medium for 1 h at room temperature and in a 2:1 mixture of embedding medium to propylene oxide at room temperature overnight. Finally, the mixture was replaced with 100% embedding medium for 2 h at room temperature. The embedding was completed by moving the cells into EMS embedding capsules (EMS) and filling with the embedding medium. The capsules were incubated in oven at 60°C for 24 h to generate blocks. After cooling to room temperature, the blocks were cut and ultra-thin sections of 100–200 nm were produced using an ultramicrotome (RMC Boeckeler Instruments, Inc., Tucson, AZ, United States). Sections were then loaded on 400 mesh Formvar/Carbon-supported copper grids. After drying, the cells were inspected by transmission electron microscopy (JEOL-JEM 1400) operating at 120 kV.

### Protein Extraction for Proteomic Study

Proteins were extracted from triplicate sets of ∼2 × 10^6^ cells/mL for each patient and the normal control using lysis buffer (0.5 mL, pH 8.8, 30 mM Tris buffer containing 7 M urea, 2 M thiourea, 4% Chaps, 1× protease inhibitor mix). The lysate was slowly vortexed for 1 h at room temperature and then sonicated using the Microsonicator (Qsonica Sonicators, Newtown, CT, United States) with a 30% pulse and two intervals of 1 min separated by 1 min each on ice. Dithiothreitol (DTT, 50 mM) was then added and the protein lysates were centrifuged at 20,000 × *g* for 1 h at 4°C. The pellets were then removed and the solubilized proteins in the supernatants were precipitated using a 2D clean-up kit, according to the manufacturer’s protocol (GE Healthcare, Chicago, IL, United States).

### Protein Labeling Using Cyanine

The protein pellets were solubilized in labeling buffer (7 M Urea, 2 M Thiourea, 30 mM Tris–HCl, and 4% CHAPS, pH 8.5). Insoluble material was pelleted by centrifugation (12,000 × *g*, room temperature, 5 min), and protein concentrations were measured in triplicate using the 2D-Quant Kit (GE Healthcare), and the pH values of the samples were adjusted to 8.5 using NaOH (100 mM). The proteins were then labeled (400 pmol CyDyeTM DIGE Fluor; GE Healthcare) in 1 μL of DMF and mixed with 50 μg of protein. The mixtures were incubated on ice for 30 min in a dark place. Afterward, the labeling reaction was terminated by adding 1 μL of 10 mM lysine. A fluorophore was used to covalently label each technical duplicate of the samples. These fluorophores were either Cy3 or Cy5. A mixture of equal amounts of protein isolated from each sample in the assay was labeled with Cy2 and used as an internal standard.

### Two-Dimensional Electrophoresis, Image Scanning, and Preparative Gel

Six Immobiline Dry Strips (24 cm, pH 3–11; GE Healthcare, Upsala, Sweden) were passively rehydrated (30 V, 12 h). This was followed by isoelectric focusing using an Ettan IPGphor IEF unit (GE Healthcare). Focusing was performed at 20°C and 50 μA per strip, according to the following sequence: 500 V for 1 h, 1000 V for 1 h, 8000 V for 3 h, 8000 V up to a total of 45,000 Vh. After the first dimension, the strips were equilibrated and separated on 12.5% (SDS-PAGE) gels using an Ettan Dalt Six device (GE Healthcare). The gels were scanned using a Typhoon 9400 scanner (GE Healthcare) with appropriate wavelengths and filters for Cy2, Cy3, and Cy5 dyes. Total protein (1 mg) was obtained from a pool of equal protein amounts for each sample. This was denatured in lysis buffer and mixed in a rehydration buffer. Gels were then stained by Colloidal Coomassie Blue.

### Protein Identification by MALDI-TOF Mass Spectrometry

The spots on Coomassie-stained gels were manually excised, washed, and digested according to a previously published protocol (Alfadda et al., 2013). The mixture of tryptic peptides (1 μL) derived from each protein was spotted onto a MALDI target (384 AnchorChip MTP 800 μm; Bruker Daltonics, Bremen, Germany) together with 0.8 μL of matrix (10 mg of α-cyano-4-hydroxycinnamic acid (CHCA) in 1 μL of 30% CH3CN and 0.1% TFA) and then left to dry at room temperature before mass spectrometry (MS). Spectra were generated using a MALDI-TOF MS (UltraFlexTrem, Bruker Daltonics) in the positive mode with a target voltage of 25 kV and pulsed ion extraction voltage of 20 kV. The reflector voltage was set to 21 kV and detector voltage was set to 17 kV. Peptide mass fingerprints (PMF) were calibrated against a standard (Peptide Calibration Standard II, Bruker Daltonics). The PMFs were processed using Flex Analysis (v. 2.4, Bruker Daltonics). The MS data were interpreted using BioTools v. 3.2 (Bruker Daltonics) and the MASCOT search algorithm (v. 2.0.04 updated 09/05/2017; Matrix Science Ltd., London, United Kingdom). MASCOT search parameters were as follows: fixed propionamide modification of cysteine, oxidation of methionine as a variable modification, one missed cleavage site (e.g., in the case of incomplete trypsin hydrolysis), and a mass tolerance of 100 ppm. Identified proteins were accepted if they showed a MASCOT score of greater than 56 and *P* < 0.05. Not all spots of interest could be identified owing to a low abundance or insufficient intensity; other spots were mixtures of multiple proteins.

### Image Acquisition and Analysis

DIGE images were analyzed using Progenesis SameSpots v. 3.3 (Non-linear Dynamics Ltd., Newcastle upon Tyne, United Kingdom). First, images were aligned. Prominent spots were used to manually assign 45 vectors to digitized images within each gel and the automatic vector tool was used to add additional vectors (390 total vectors), which were manually revised and edited, if necessary. These vectors were used to warp and align gel images against a reference image of one internal standard across and within each gel. Groups were established according to the experimental design and spot-normalized volumes were used to select statistically significant spots. The normalized volume of each spot on each gel was calculated from the Cy3 (or Cy5)-to-Cy2 spot volume ratio. The software performed log transformation of the spot volumes to generate normally distributed data. Log-normalized volumes were used to quantify differential expression. Independent direct comparisons were made between the VLCAD and control groups and fold differences and P-values were calculated using one-way ANOVA. All spots were pre-filtered and manually checked before applying the statistical criteria (ANOVA, *P* ≤ 0.05 and fold change ≥ 1.5). Normalized spot volumes, instead of spot intensities, were used for statistical processing. Only those spots that fulfilled the abovementioned statistical criteria were included in the MS analysis. In addition, sample expression profiles were separated into clusters. Each cluster shows a spot that is representative of the abundance across gels. This method was used to detect protein upregulation in patients with a VLCAD deficiency using Progenesis SameSpots.

### Pathway Analysis

A pathway analysis was performed by importing the quantitative data into Ingenuity Pathway Analysis (IPA) (Ingenuity^®^ Systems)^1^. This software aids in determining the functions and pathways that are most strongly associated with the protein list by overlaying the experimental expression data on networks constructed from published interactions.

### Statistical Analysis

All experiments were repeated three times independently, unless otherwise stated. P-values of less than 0.05 indicated significant. Student’s *t*-tests and ANOVA were used, as appropriate.

## Data Availability Statement

All datasets generated for this study are included in the article/Supplementary Material.

## Ethics Statement

The studies involving human participants were reviewed and approved by the King Abdullah International Medical Research Center IRB Committee, Ministry of National Guard Health Affairs. Written informed consent to participate in this study was provided by the participants’ legal guardian/next of kin.

## Author Contributions

AhA, MA, SAG, and BMA designed the experiments. AhA, MA, and MAB screened the patients. MAB, AhA, and SA collected the samples and validated sequencing data. AhA, MA, RA, SM, AhM, AfM, HB, SAG, and BMA wrote the manuscript. BA, SA, AmA, SAM, RA, HB, AfM, and AhM performed the experiments. AhA, SA, AmA, and BMA validated the results. AhA, MA, SM, HB, AfM, and BMA interpreted the data. AhA, IA, AsA, and AfM performed proteomic analysis. BMA supervised the project and funded the experiments.

## Conflict of Interest

The authors declare that the research was conducted in the absence of any commercial or financial relationships that could be construed as a potential conflict of interest.

## Footnotes

1 http://www.ingenuity.com

## Supplementary Material

FIGURE S1 Representative fluorescent protein profiles by 2D-DIGE. **(A)** Control sample labeled with Cy3. **(B)** VLCAD sample labeled with Cy5. **(C)** Pooled internal control labeled with Cy2. **(D)** Representative overlay of Cy3/Cy5 images.

FIGURE S2 Very-long-chain acyl-coenzyme A dehydrogenase deficiency expression profiles compared to those of controls. The profiles are separated into clusters of expression patterns; the number of spots for each cluster are indicated. Each line represents the standardized abundance of a spot across all gels and belongs to one of the clusters generated by a hierarchical cluster analysis. The spots with increased abundance indicate the 37 proteins that were upregulated in patients with VLCAD deficiency (Progenesis SameSpots).

FIGURE S3 Successful differentiation of mesenchymal stem cells into neurons using both Hs27 cell line and patient primary cells, BA28 and BA38. Neural morphological structure can be seen clearly on differentiated cells compared to original shape.

FIGURE S4 Flow cytometry showing isolation of positive CD90 population with has been analyzed with CD105 which was also analyzed for CD144. The CD90 positive cells were also positive for CD105 and CD144.

FIGURE S5 Immunocytochemistry assay showing GFAP positive neurons after differentiation.

FIGURE S6 Immunocytochemistry assay showing nestin positive neurons after differentiation.

TABLE S1 List of significant differentially expressed proteins identified in the cells between control vs. VLCAD using 2D-DIGE with differences in fold change. Protein name, accession number, Mascot score, MS % coverage, protein MW, and pI values according to Uniprot database are listed.
