# Supplementary figures and images for: Proteomic and Molecular Assessment of the Common Saudi Variant in ACADVL Gene Through Mesenchymal Stem Cells
Source: Front Cell Dev Biol. 2020 Jan 10;7:365. doi: 10.3389/fcell.2019.00365 (PMC6979051; doi:10.3389/fcell.2019.00365)

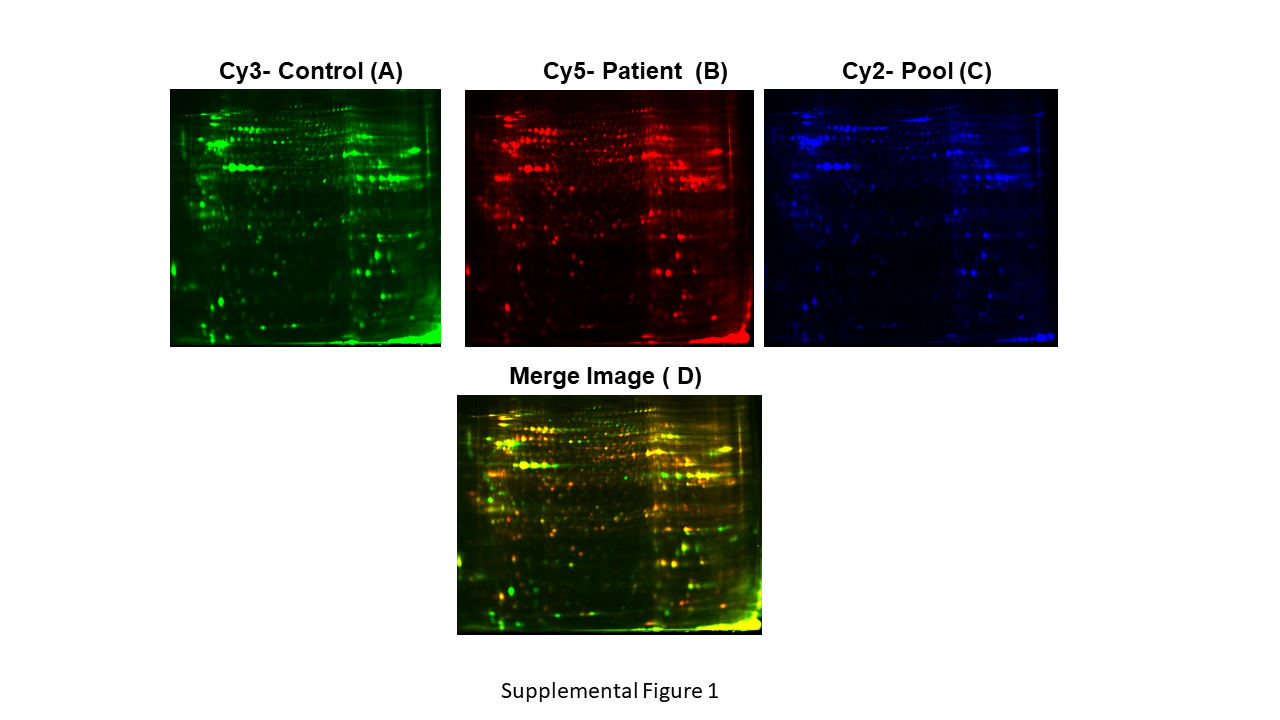

Supplement: FIGURE S1 — Representative fluorescent protein profiles by 2D-DIGE. (A) Control sample labeled with Cy3. (B) VLCAD sample labeled with Cy5. (C) Pooled internal control labeled with Cy2. (D) Representative overlay of Cy3/Cy5 images. [file Image_1.TIF]

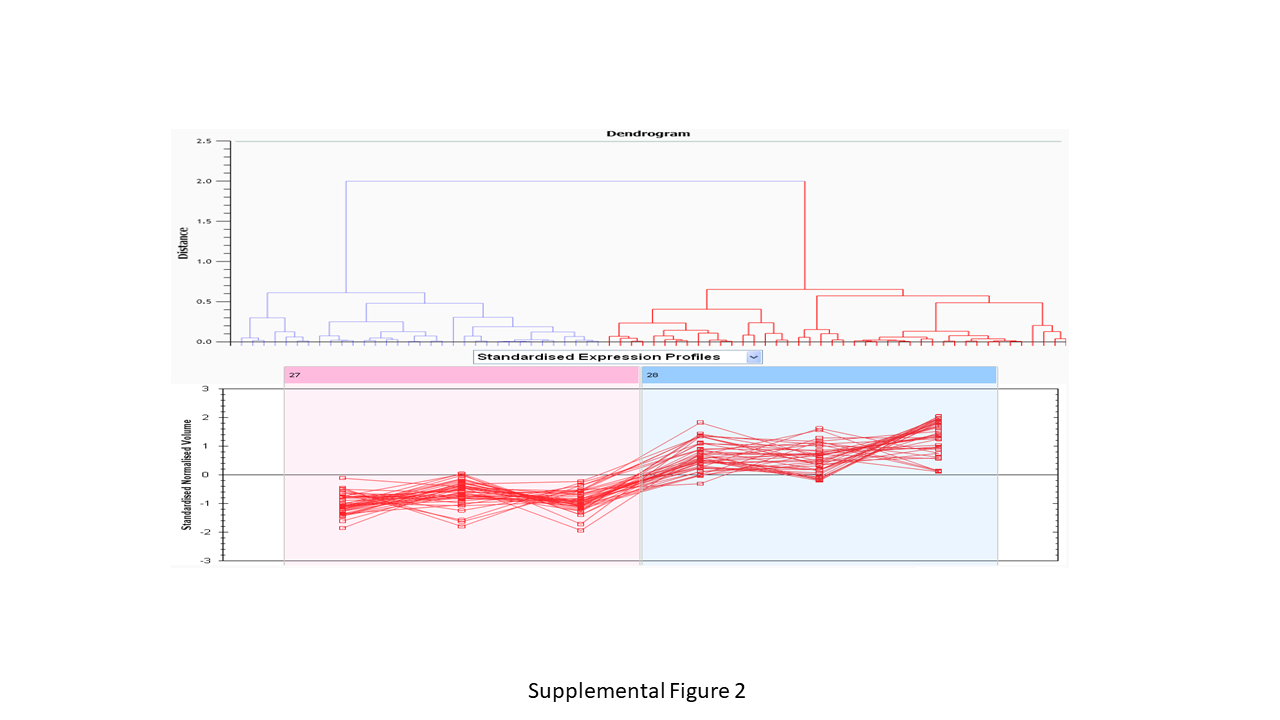

Supplement: FIGURE S2 — Very-long-chain acyl-coenzyme A dehydrogenase deficiency expression profiles compared to those of controls. The profiles are separated into clusters of expression patterns; the number of spots for each cluster are indicated. Each line represents the standardized abundance of a spot across all gels and belongs to one of the clusters generated by a hierarchical cluster analysis. The spots with increased abundance indicate the 37 proteins that were upregulated in patients with VLCAD deficiency (Progenesis SameSpots). [file Image_2.TIF]

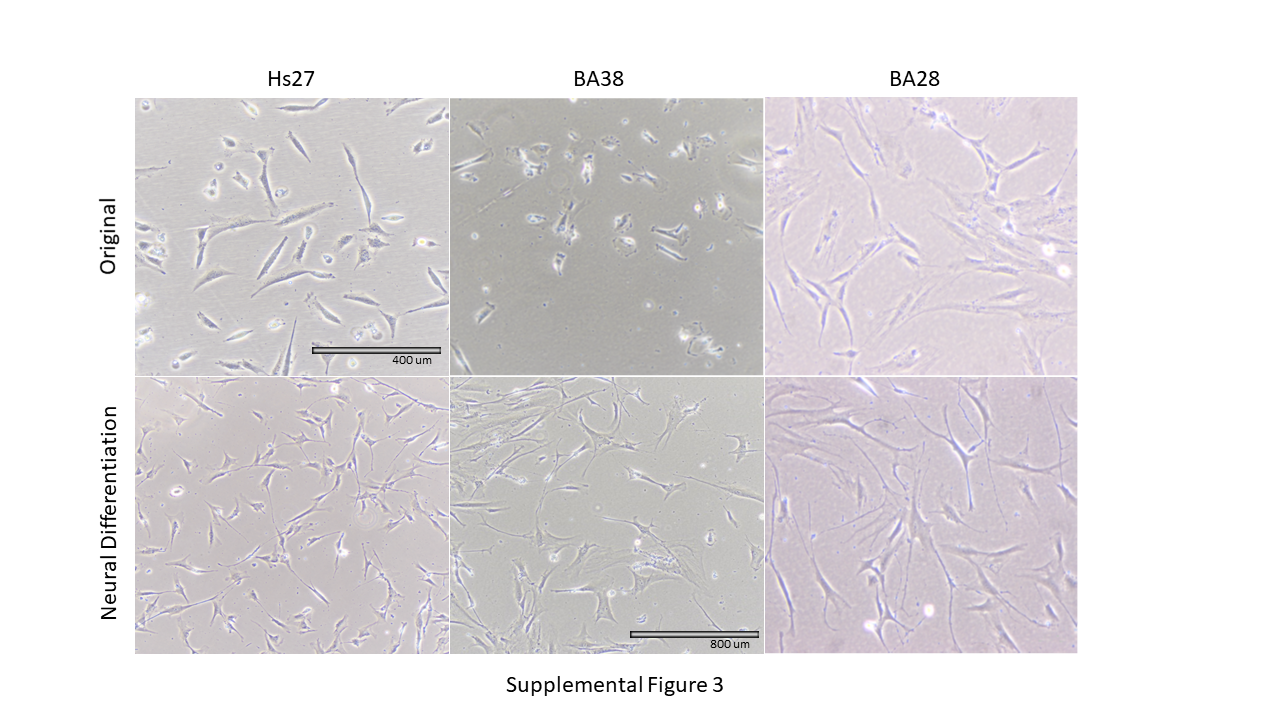

Supplement: FIGURE S3 — Successful differentiation of mesenchymal stem cells into neurons using both Hs27 cell line and patient primary cells, BA28 and BA38. Neural morphological structure can be seen clearly on differentiated cells compared to original shape. [file Image_3.TIF]

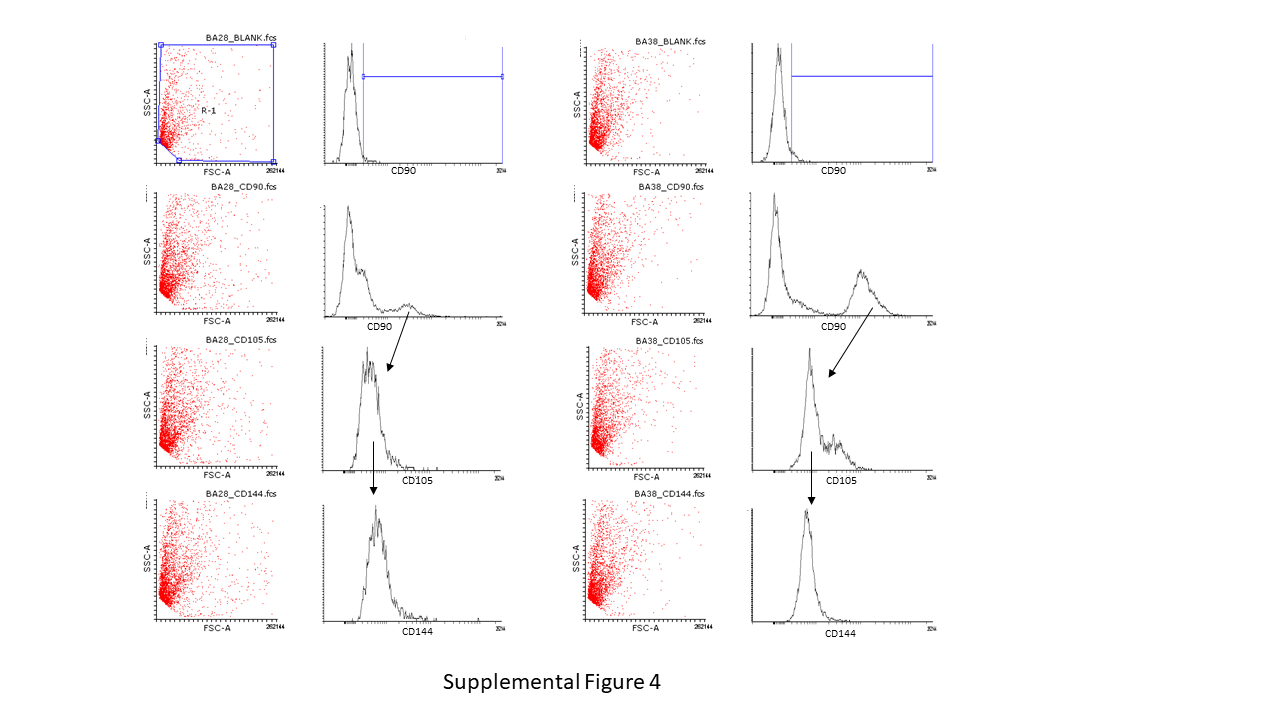

Supplement: FIGURE S4 — Flow cytometry showing isolation of positive CD90 population with has been analyzed with CD105 which was also analyzed for CD144. The CD90 positive cells were also positive for CD105 and CD144. [file Image_4.TIF]

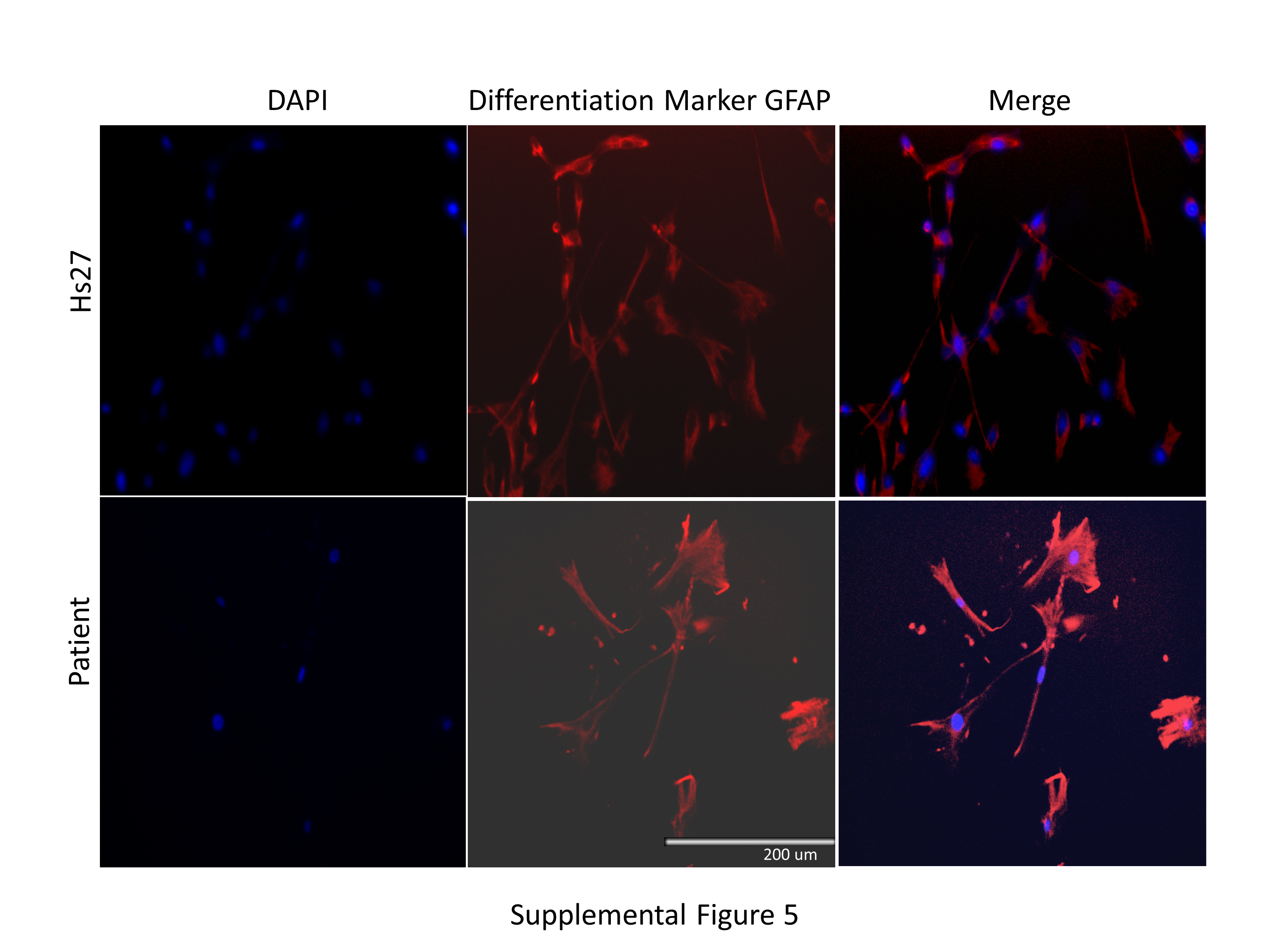

Supplement: FIGURE S5 — Immunocytochemistry assay showing GFAP positive neurons after differentiation. [file Image_5.TIF]

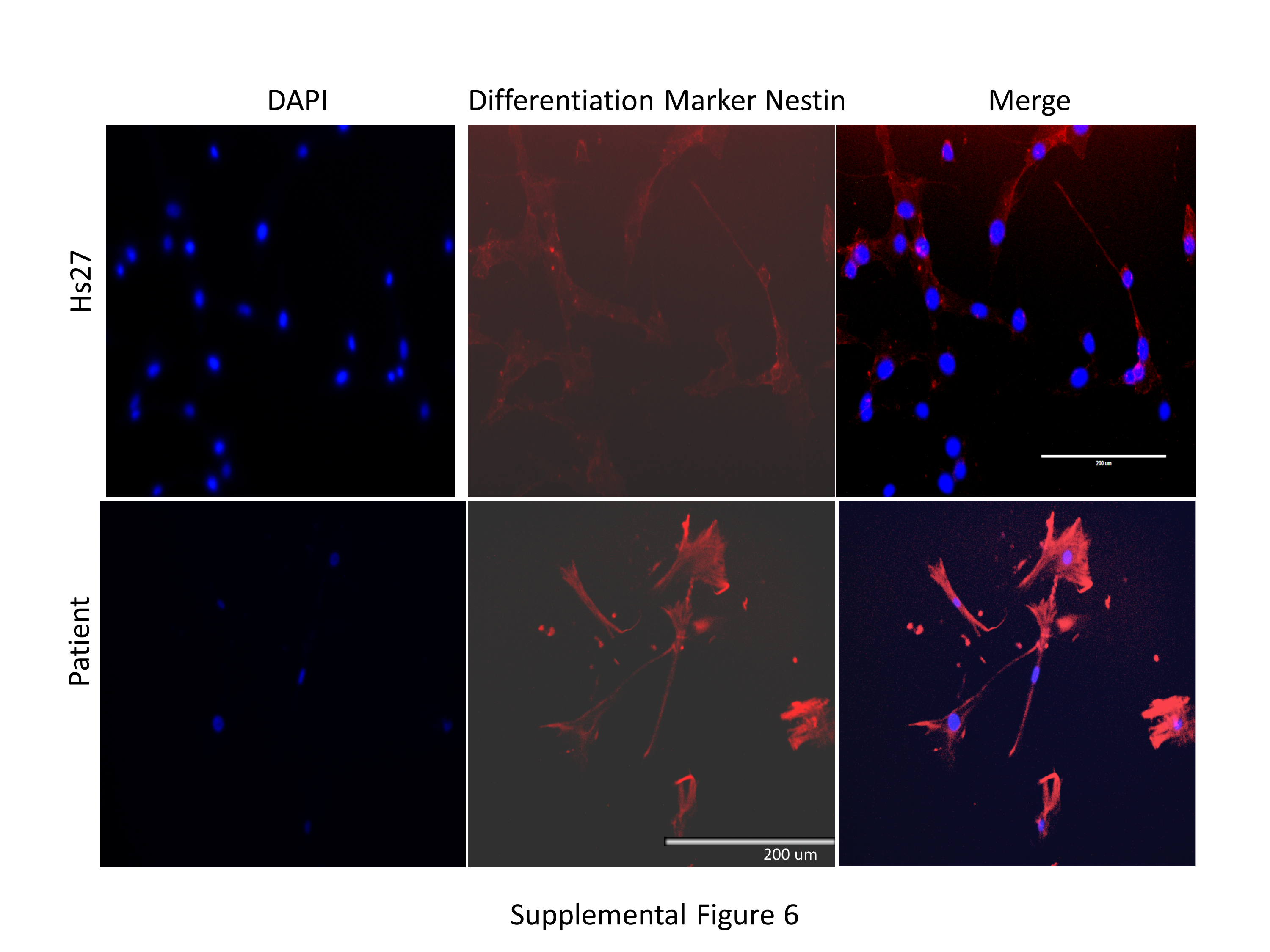

Supplement: FIGURE S6 — Immunocytochemistry assay showing nestin positive neurons after differentiation. [file Image_6.TIF]
