# Supplementary material for: Proteomic and Molecular Assessment of the Common Saudi Variant in ACADVL Gene Through Mesenchymal Stem Cells
Source: Front Cell Dev Biol. 2020 Jan 10;7:365. doi: 10.3389/fcell.2019.00365 (PMC6979051; doi:10.3389/fcell.2019.00365)
Supplement: TABLE S1 — List of significant differentially expressed proteins identified in the cells between control vs. VLCAD using 2D-DIGE with differences in fold change. Protein name, accession number, Mascot score, MS % coverage, protein MW, and pI values according to Uniprot database are listed. [file Table_1.docx]

**Supplemental TABLE 1:** List of significant differentially expressed proteins identified in the cells between control vs VLCAD using 2D-DIGE. With differences in fold change. Protein name, accession number, Mascot score, MS % coverage, protein MW and pI values according to Uniprot database are listed.

| Spot No | Accession No | **Protein Name** | MASCOT ID | Pi | MW | Cov % | Score |
| --- | --- | --- | --- | --- | --- | --- | --- |
| 1018 | P04406 | **Glyceraldehyde-3-phosphate dehydrogenase** | G3P_HUMAN | 8.57 | 36201 | 58 | 111 |
| 1594 | Q6XPS3 | **Phosphotidylinositol 3,4,5-triphosphate 3-phaosphate** | TPTE2_HUMAN | 8.1 | 61472 | 29 | 56 |
| 1478 | P22392 | **Nucleoside diphosphate kinase B** | NDKB_HUMAN | 8.52 | 17401 | 62 | 70 |
| 748 | P14618 | **Pyruvate kinase PKM** | KPYM_HUMAN | 7.96 | 58470 | 42 | 150 |
| 1428 | P63241 | **Eukaryotic translation initiation factor 5A-1** | IF5A1_HUMAN | 5.08 | 17049 | 22 | 56 |
| 552 | P38646 | **Stress-70 protein, mitochondrial** | GRP75_HUMAN | 5.87 | 73980 | 34 | 103 |
| 692 | Q99733 | **Nucleosome assembly protein 1-like 4** | NP1L4_HUMAN | 4.6 | 42568 | 34 | 71 |
| 1438 | P63241 | **Eukaryotic translation initiation factor 5A-1** | IF5A1_HUMAN | 3.87 | 73920 | 113 | 60 |
| 620 | Q9BY41 | **Histone deacetylase 8** | HDAC8_HUMAN | 5.48 | 70894 | 54 | 230 |
| 819 | P43686 | **26S proteasome regulatory subunit 6B** | PRS6B_HUMAN | 5.09 | 47431 | 44 | 97 |
| 446 | Q9P1Z9 | **Coiled-coil domain-containing protein 180** | CC180_HUMAN | 5.74 | 192404 | 11 | 57 |
| 670 | P12270 | **Nucleoprotein TPR** | TPR_HUMAN | 4.97 | 267530 | 12 | 60 |
| 703 | P07437 | **Tubulin beta chain** | TBB5_HUMAN | 4.78 | 50695 | 52 | 210 |
| 707 | P07237 | **Protein disulfide-isomerase** | PDIA1_HUMAN | 4.76 | 57480 | 43 | 133 |
| 1589 | P62805 | **Histone H4** | H4_HUMAN | 11.36 | 11360 | 53 | 91 |
| 508 | P08238 | **Heat shock protein** | HS90B_HUMAN | 4.97 | 83539 | 29 | 79 |
| 1017 | P04406 | **Glyceraldehyde-3-phosphate dehydrogenase** | G3P_HUMAN | 8.57 | 36201 | 38 | 57 |
| 1016 | P06748 | **Nucleophosmin** | NPM_HUMAN | 4.44 | 3274 | 39 | 67 |
| 913 | P63261 | **Actin, cytoplasmic 2** | ACTG_HUMAN | 5.31 | 42108 | 42 | 85 |
| 802 | Q14204 | **Cytoplasmic dynein 1 heavy chain 1** | DYHC1_HUMAN | 5.01 | 534809 | 8 | 56 |
| 708 | P10809 | **60 kDa heat shock protein, mitochondrial** | CH60_HUMAN | 5.7 | 61187 | 46 | 188 |
| 1328 | P48047 | **ATP synthase subunit O, mitochondrial** | ATPO_HUMAN | 9.97 | 23377 | 52 | 81 |
| 704 | P10809 | **60 kDa heat shock protein, mitochondrial** | CH60_HUMAN | 5.7 | 61187 | 52 | 171 |
| 1608 | Q9NZJ6 | **Ubiquinone biosynthesis O-methyltransferase, mitochondrial** | COQ3_HUMAN | 7.12 | 414481 | 21 | 56 |
| 812 | P68104 | **Elongation factor 1-alpha 1** | EF1A1_HUMAN | 9.1 | 50451 | 37 | 104 |
| 586 | P13639 | **Elongation factor 2** | EF2_HUMAN | 6.91 | 96246 | 27 | 59 |
| 1421 | P35908 | **Keratin, type II cytoskeletal 2 epidermal** | K22E_HUMAN | 8.07 | 65678 | 30 | 89 |
| 723 | P25705 | **ATP synthase subunit alpha, mitochondrial** | ATPA_HUMAN | 9.16 | 59828 | 56 | 170 |
| 1491 | Q8J015 | **60S ribosomal protein L13a** | Q8J015_HUMAN | 9.48 | 17979 | 62 | 69 |
| 530 | P13639 | **Elongation factor 2** | EF2_HUMAN | 6.14 | 96246 | 47 | 217 |
| 281 | Q14697 | **Neutral alpha-glucosidase AB** | GANAB_HUMAN | 5.74 | 107263 | 30 | 287 |
| 835 | P68104 | **Elongation factor 1-alpha 1** | EF1A1_HUMAN | 9.1 | 50451 | 32 | 68 |
| 937 | P63261 | **Actin, cytoplasmic 2** | ACTG_HUMAN | 5.31 | 42108 | 66 | 149 |
| 1136 | Q07021 | **Complement component 1 Q subcomponent-binding protein, mitochondrial** | C1QBP_HUMAN | 4.74 | 31742 | 51 | 62 |
| 1411 | P00441 | **Superoxide dismutase [Cu-Zn]** | SODC_HUMAN | 5.7 | 15154 | 68 | 78 |
| 622 | P11142 | **Heat shock cognate 71 kDa protein** | HSP7C_HUMAN | 5.37 | 71082 | 27 | 57 |
| 1324 | Q06830 | **Peroxiredoxin-1** | PRDX1_HUMAN | 8.27 | 22324 | 60 | 90 |
| 435 | P53396 | **ATP-citrate synthase** | ACLY_HUMAN | 6.95 | 121674 | 18 | 67 |
| 1610 | P12883 | **Myosin-7** | MYH7_HUMAN | 5.63 | 223757 | 15 | 58 |
| 1012 | P06748 | **Nucleophosmin** | NPM_HUMAN | 4.64 | 32766 | 33 | 36 |
| 671 | P14618 | **Pyruvate kinase PKM** | KPYM_HUMAN | 7.96 | 38470 | 49 | 164 |
| 598 | P11142 | **Heat shock cognate 71 kDa protein** | HSP7C_HUMAN | 5.37 | 71082 | 46 | 164 |
| 1041 | P52907 | **F-actin-capping protein subunit alpha-1** | CAZA1_HUMAN | 5.45 | 33673 | 53 | 88 |
| 1347 | Q8WVF1 | **Protein OSCP1** | SCP1_HUMAN | 5.71 | 43306 | 43 | 62 |
| 1014 | P07910 | **Heterogeneous nuclear ribonucleoproteins C1/C2** | HNRPC_HUMAN | 4.91 | 33707 | 37 | 60 |
| 1300 | Q9UI30 | **Multifunctional methyltransferase subunit TRM112-like protein** | TR112_HUMAN | 5.21 | 14304 | 63 | 68 |
| 1318 | Q9UI30 | **Multifunctional methyltransferase subunit TRM112-like protein** | TR112_HUMAN | 5.21 | 14304 | 47 | 57 |
| 1354 | P07741 | **Adenine phosphoribosyl transferase** | APT_HUMAN | 5.78 | 19766 | 82 | 144 |
| 1328 | Q96KK5 | **Histone H2A type 1-H** | H2A1H_HUMAN | 10.88 | 13898 | 66 | 75 |
| 737 | P14618 | **Pyruvate kinase PKM** | KPYM_HUMAN | 7196 | 58470 | 44 | 153 |
| 353 | P12883 | **Myosin-7** | MYH7_HUMAN | 5.13 | 223717 | 11 | 63 |
| 913 | P08670 | **Vimentin** | VIME_HUMAN | 5.06 | 53676 | 61 | 166 |
| 1071 | Q13347 | **Eukaryotic translation initiation factor 3 subunit I** | EIF3I_HUMAN | 5.38 | 36878 | 53 | 85 |
| 419 | P54709 | **Sodium/potassium-transporting ATPase subunit beta-3** | T1B3_HUMAN | 5.26 | 56526 | 45 | 119 |
| 814 | Q9Y230 | **RuvB-like 2** | RUVB2_HUMAN | 5.99 | 51296 | 47 | 136 |
| 751 | P08670 | **Vimentin** | VIME_HUMAN | 5.06 | 53676 | 67 | 219 |
| 841 | P06576 | **ATP synthase subunit beta, mitochondrial** | ATPB_HUMAN | 5.26 | 56526 | 67 | 267 |

- 1. **Differentiation experiment**

All cells have been culture in neurobasal media (goibco; 21103049) supplemented with 20% DMEM/F12 (gibco; 12634010), B-27 Supplement minus Vitamin A (gibco; 12587010), and 10% FBS (9%, gibco; 10100147 and 1% atcc: acs-3002). Cells were maintained in the same media with continuous passaging and media changing for one month. Cells have been examined morphologically and neural unique structures were detected, supplemental Fig. 3.

- 1. **Flow cytometry experiment**

All antibody markers CD90 (IM1839U), CD105 (A07414), and CD144 were purchased from Beckman Coulter, USA, supplemental Fig. 4. Flow cytometry procedure has been described previously. (Alshareeda et al. 2018)

Alshareeda AT, Rakha E, Alghwainem A, Alrfaei B, Alsowayan B, Albugami A, Alsubayyil AM, Abomraee M, Mohd Zin NK. 2018. The effect of human placental chorionic villi derived mesenchymal stem cell on triple-negative breast cancer hallmarks. PLOS ONE.13:e0207593.
